# “Internal fixation of proximal humeral fractures using the Polarus intramedullary nail: our institutional experience and review of the literature”

**DOI:** 10.1186/1749-799X-7-39

**Published:** 2012-12-19

**Authors:** Peter V Giannoudis, Fragiskos N Xypnitos, Rozalia Dimitriou, Nick Manidakis, Roger Hackney

**Affiliations:** 1 Department of Trauma and Orthopaedics, School of Medicine, University of Leeds, Leeds General Infirmary, Clarendon wing Level A, Great George Street, LS1 3EX, Leeds, UK

**Keywords:** Proximal humeral fractures, Polarus nail, Union, Complications, Functional outcome

## Abstract

**Background:**

The purpose of this study is to evaluate the functional outcome, union and complication rates after surgical treatment of unstable or displaced proximal humeral fractures using the Polarus intramedullary nail, by reviewing our institutional experience and the relevant current literature.

**Methods:**

Twenty-seven patients were treated operatively for proximal humeral fracture using the Polarus nail. Fractures were classified according to Neer’s classification. A number of parameters including patient demographics, mechanism of injury, operative time, time to union and complications were recorded. Functional outcome was evaluated using the Constant Shoulder Score. A comparison among functional outcomes in patients >60 years in relation to the younger ones was performed. Moreover, a review of the literature was carried out to evaluate the overall union and complication rates.

**Results:**

Two patients lost to follow-up were excluded from the analysis. For the twenty-five patients (mean age: 61 years), the mean follow-up was 36 months. There were 7 complications (28%), including one fixation failure, four protruded screws, one superficial infection and one case of impingement. The union rate was 96% (mean time to union: 4.2 months). The mean Constant score was 74.5 (range: 48–89). Patients under the age of 60 had a better functional outcome compared to patients >60 years of age (*p*<*0*.*05*). From the literature review and from a total of 215 patients treated with a Polarus nail, the mean union rate was 95.8%, the overall reported complication rate, including both minor and major complications, ranged widely from 9.3% up to 70%.

**Conclusions:**

The Polarus nail was found to be an effective implant for stabilisation of proximal humeral fractures. Functional outcome is for the vast majority of the cases excellent or good, but in elderly patients a lower Constant score can be expected.

## Introduction

Proximal humeral fractures account for 5% of all fractures and constitute the third most frequent fracture in elderly patients, with substantial economic effect on health care systems [1-4]. The restoration of a painless shoulder with satisfactory function is the goal of treatment. Non-operative treatment can be applied for most of the stable proximal humeral fractures with minimal or no displacement with good results [2]. A fracture is considered to be displaced if the fracture fragment has a displacement more than 1 cm or an angulation more than 45° in at least one view of the trauma-series radiographs [5].

Non-operative treatment of unstable or displaced proximal humeral fractures may result in malunion and stiffness of the shoulder [2,5]. Different types of internal fixation have been developed for the surgical treatment of these fractures including plates and screws, staples, wires, percutaneous pinning and intramedullary nails [6-9]. Currently, for three and especially four-part fractures there is a trend to proceed with shoulder hemiarthroplasty [4]. All the aforementioned operative fixation techniques have demonstrated different outcomes and complication rates [6-13]; and this diversity of options implies that there is an ongoing effort to find out what is the best osteosynthesis technique to stabilise certain fracture patterns.

The Polarus intramedullary nail (Acumed, Inc, Beaverton, OR, USA) is one of the available locked antegrade intramedullary (IM) devices for the surgical treatment of proximal humeral fractures and it allows screw stabilisation of the humeral head and tuberosities. There are scarce reports in the literature reporting on functional and radiological outcome and complication rate after its clinical use [10,14-20].

In the present study, our aim is to report on our clinical experience from the surgical treatment of unstable or displaced proximal humeral fractures using this intramedullary device. Moreover, a review of the current literature was carried out to evaluate the overall union rates and incidence of reported complications.

## Materials and methods

From January 2007 to December 2009, all consecutive patients that were treated in our institution with the Polarus nail for stabilisation of fractures of the proximal humerus were included in this retrospective study. Institutional board approval was obtained. Using true anteroposterior (AP) and y-views radiographs of the shoulder and, when necessary, CT scan to accurately assess the fracture pattern (in cases of comminuted fracture patterns), fractures were classified according to Neer’s classification [5]. Inclusion criteria were displaced 2-part and 3-part fractures as well as some 4-part fractures where satisfactory reduction was possible by closed means. Exclusion criteria for the use of the Polarus nail were minimally displaced or undisplaced fractures of the proximal humerus, severely comminuted 4-part or 3-part fractures (especially in elderly patients with poor bone quality), head split fractures, fracture-dislocations, and impaction fractures with involvement of more than 40% of the articular surface. Osteoporosis and arthritis of the glenohumeral joint were also factors that were taken into account. In these cases, we used other fixation devices or hemiarthroplasty.

In all cases, a short Polarus nail was used. This device is a cannulated locked antegrade intramedullary humeral rod with a tapered profile to reduce distal stress concentration, and it has a spiral array of four 5-mm proximal screw-holes and two 3.5-mm distal interlocking holes [10,14,16,17].

Passive physiotherapy was initiated from the 1st postoperative day, including pendulum motion and passive elevation and rotation. At the same time, all patients were encouraged to actively exercise the wrist and elbow joints. Subsequently, active assisted exercises after the 2nd week and active motion of the shoulder joint at 4 weeks was encouraged. Patients were trained in self-assisted shoulder abduction and elevation using the uninjured extremity as an assistant. Outpatient physiotherapy was initiated 2 weeks postoperatively. Exercises under resistance were restrained until the 6th postoperatively week or until fracture union was radiologically confirmed.

Following discharge from the hospital, patients were followed up in the outpatient clinic at 2 and 6 weeks, and at 3, 6, 12 months and yearly thereafter (maximum 3 years) as indicated. Anteroposterior and axillary radiographs were taken at each follow-up in order to evaluate the progress in fracture union and the presence of implant related complications. The time to union as well as all minor or major complications and their management were documented. Numerous other parameters were recorded and analysed including patient demographics, mechanism of injury, associated injuries, days to surgery and operative time. The mean follow-up time was 36 months (range of 27–43).

The functional outcome was assessed using the Constant shoulder scoring system [21] at the final follow up. The scoring system is constituted from 4 categories of interest: pain, activities of daily leaving, range of motion and strength. Pain is scored with a maximum of 15 points (no pain) whereas the activity of daily living maximum score is 20 points (no limitations in daily living and recreational activities, no night sleep disturbance and above head usage of the arm). The maximum score for the range of movement parameter is 40 points (evaluates forward flexion, abduction, internal and external rotation) whilst power is estimated by doubling the average kilos from 5 pulls and has a maximum score of 25 points. Scores below 50% were considered to be a poor result, between 50% and 75% a satisfactory result, and scores above 75% to be an excellent result [18,20]. The strength in the shoulder was measured with an isometric tensiometer. Additionally, a comparative evaluation of the functional outcomes in patients older than 60 years in relation to the younger ones was performed using the Mann–Whitney *U*-test. Statistical analysis was performed using SPSS version 13.00 (Statistical Package for the Social Sciences, SPSS Inc., Chicago, Ill., USA) and a p-value of 0.05 or less was considered as statistically significant.

Finally, the current literature was reviewed and all relevant studies reporting on the clinical use of this fixation method were collected. Data regarding total number and mean age of patients, fracture patterns, union, functional outcome and scoring system used as well as complications rate were documented and analysed.

### Ethical approval

Institutional board approval was obtained from the local Trauma & Orthopaedic Directorate and the research carried out was in compliance with the Helsinki Declaration. Every patient consented to participate in this study for publication of this report and any accompanying images.

## Results

Out of 27 patients that met the inclusion criteria, 2 patients were lost to follow up and therefore were excluded from the final analysis. From the remaining twenty-five patients, there were 18 women and 7 men, with a mean age of 50 years (range: 16–81) and 66 years (range: 33–92), respectively. The overall patients’ mean age was 61 years (range: 18–92). Mechanisms of injury included 18 simple falls, 5 pedestrian versus automobile impacts, and other causes in 2 cases. Three patients presented with other associated injuries as shown in Table 1. Fracture patterns included 16 2-part fractures, five 3-part and four 4-part proximal humeral fractures, and all fractures were closed.

**Table 1:** **A summary of fracture type**, **reduction**, **associated injuries**, **complications and clinical outcome for the cases included in the analysis**

**Patient**	**Fracture type**	**Reduction**	**Associated injuries**	**Complications**	**Clinical outcome**
1	2-part (SN)	Closed	None	Protruded screw	Union
2	2-part (SN)	Closed	Pneumo-haemothorax	-	Union
3	2-part (SN)	Open	-	-	Union
4	2-part (SN)	Closed	-	-	Union
5	2-part (SN)	Closed	-	Protruded screw	Union
6	2-part (SN)	Closed	-	-	Union
7	3-part (SN + GT)	Open	-	-	Union
8	4-part (SN + GT + LT)	Closed	-	Protruded screw	Union
9	2-part (SN)	Closed	-	-	Union
10	2-part (SN)	Open	-	-	Union
11	2-part (SN)	Closed	-	-	Union
12	4-part (SN + GT + LT)	Closed	-	-	Union
13	2-part (SN)	Closed	-	Protruded screw	Non-Union
14	2-part (SN)	Closed	Temporal Lobe Contusion + Supramalleolar Tibial Fracture	Superficial Infection	Union
15	2-part (SN)	Open	-	-	Union
16	2-part (SN)	Closed	Midshaft tibial fracture	-	Union
17	2-part (SN)	Closed	-	Fixation Failure	Hemi – arthroplasty
18	2-part (SN)	Open	-	-	Union
19	3-part (SN + GT)	Closed	-	Impingement	Union
20	3-part (SN + LT)	Open	-	-	Union
21	2-part (SN)	Closed	-	-	Union
22	3-part (SN + LT)	Closed	-	-	Union
23	3-part (SN + GT)	Closed	-	-	Union
24	4-part (SN + GT + LT)	Open	-	-	Union
25	4-part (SN + GT + LT)	Closed	-	-	Union

(SN: surgical neck, GT: greater tuberosity, LT: lesser tuberosity).

**Figure 1:**
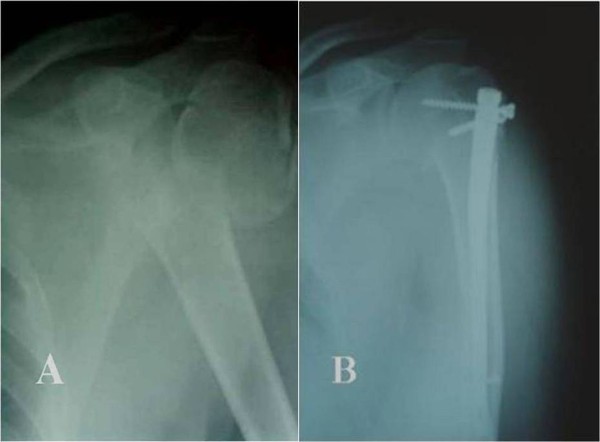
**A.A displaced 2-part proximal humeral fracture (A) treated with a short Polarus nail (B).**

**Figure 2:**
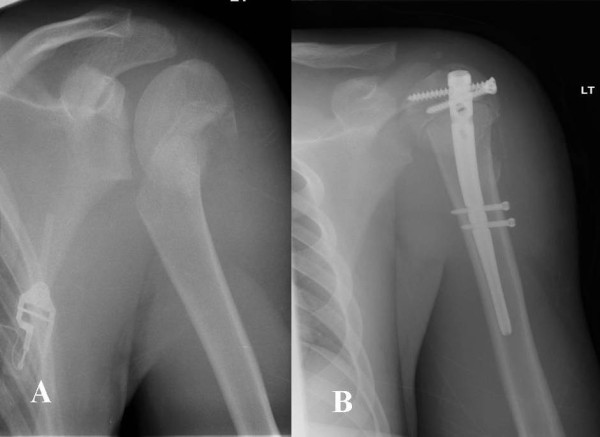
**A displaced 2-part proximal humeral fracture in a 16 year-old boy (A) treated with a short Polarus nail (B).**

**Figure 3:**
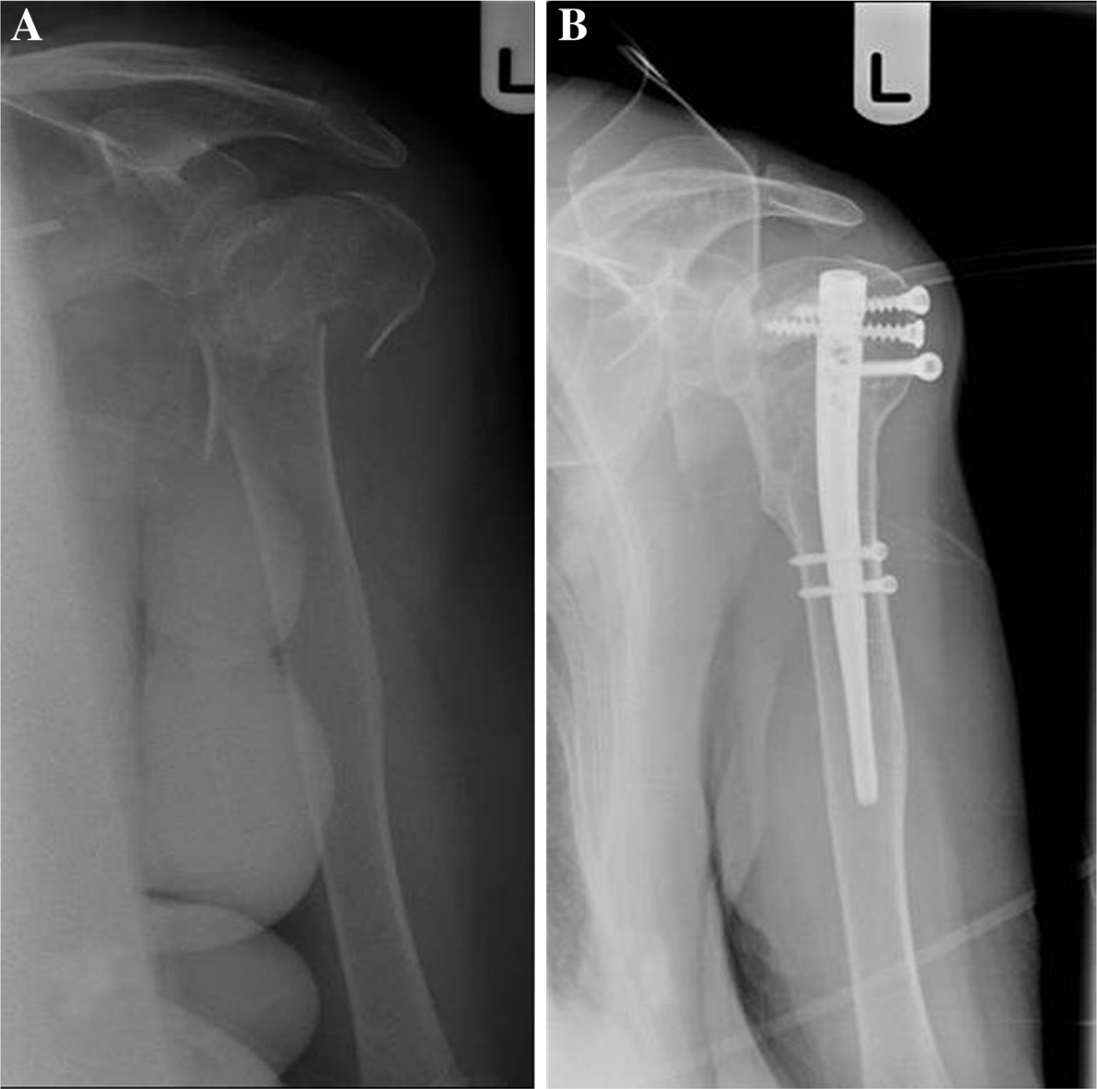
**A displaced 3-part proximal humeral fracture (A) treated with a short Polarus nail (B).** The backed-out proximal screw was removed.

The fractures were stabilised either acutely or after failure of conservative management in three cases (Figures 1, 2 and 3). Primary surgery was delayed at a median interval of 7 days (range: 0–23). The mean operative time was 51 min (range: 42–79 min). In 18 of the 25 fractures closed reduction was successful, but mini-open reduction was required in 7 cases (Table 1). Of them, the three cases where the ones that had failed conservative treatment. More particularly, two cases were 2-part and one was 3-part fracture; and for these fractures the mean time post injury was 4 months. The remaining four cases were acutely fixed fractures requiring mini open reduction with two 2-part, one 3-part and one 4-part fractures, with a mean time post injury of 7.5 days. Overall, no requirement for blood transfusion was recorded. All but one fractures progressed to union at a mean time of 4.2 months (range: 3–9), resulting in a 96% union rate.

The overall mean Constant score was 74.5 (range: 46–99). Fifteen patients (65%) had excellent results, eight patients (30%) had satisfactory results and two patients (5%) had poor results (Table 2). Patients under the age of 60 had better functional results with a median Constant score of 79 (mean: 80.8), while patients over 60 years of age had a median Constant score of 68 (mean: 68.2). This difference was statistically significant (p= 0.04), (Table 3). Regarding the effect of fracture type to the functional outcome, similar results were found in the median Constant scores between the different fracture patterns (Table 4).

**Table 2:** **Functional outcome** (**Constant score**) **at the 12**-**month follow**-**up**

**Functional outcome**	**Constant score**	**Number of patients**
Excellent	>75	15
Satisfactory	50-75	8
Poor	<50	2

**Table 3:** **Constant score** (**mean**) **in relation to patient**’**s age at the 12**-**month follow**-**up and recorded complications**

**Age group**	**Number of patients**	**Constant score**		**Complications**
		**mean**	**median**	
18-60 yrs	11	80.8	79	1 fixation failure
>60 yrs	14	68.2	68	4 screw back-out
				1 implant impingement
				1 superficial infection
**Total**	**25**		**p**=**0**.**04**	**7(28%)**

**Table 4:** **Constant scores** (**median and range**) **according to the type of the proximal humeral fracture**

**Fracture type**	**Number of patients**	**Constant score(median)**	**Range**
2-part	16	79	46-95
3-part	5	84	50-99
4-part	4	81	77-85

In terms of complications (Table 3), no major complications such as iatrogenic neurovascular injuries occurred, and no clinical or radiographic cases of humeral head avascular necrosis were recorded. Only one major complication encountered (4%) involving failure of the fixation in a 2-part proximal humeral fracture at 2 weeks post-operatively requiring revision surgery and conversion to shoulder hemiarthroplasty. The remaining were minor complications including one case of superficial wound infection (4%) that was treated successfully with local wound care and oral antibiotics, protrusion of one or more proximal interlocking screws in four cases (16%), and nail prominence in one case (4%). The two latter minor complications required removal of the prominent screws in three cases and nail removal after fracture’s consolidation, respectively.

Finally, the literature review revealed eight studies with a total of 215 patients that had been treated with the Polarus nail for proximal humeral fractures [10,14-20]. The overall union rate ranged from 65% to 100% for the majority of the studies, with a mean union rate of 95.8%. The overall complication rate, including both minor (superficial infection, proximal screw back-out or proximal migration, etc.) and major (AVN, non-union) complications, ranged widely from 9.3% up to 70%. All reported complications and functional outcomes with the use of different scoring systems from the reviewed studies are summarised in Table 5.

**Table 5:** **Review of all studies** (**215 patients**) **reporting on the use of Polarus nail for proximal humeral fractures**

**Author/Year**	**N of pts**	**Mean Age(years)**	**Fracture type:N of pts**	**F.U.(months)**	**Union rate**	**Time to union**	**Functional outcome(Score/mean)**	**Complications(rate)**
Georgousis [15] 2010	24	60	3-part: 13	12	100%	9.2 weeks	Constant score:	1 asymptomatic malunion
			4-part: 7			(1 delayed union that healed)	- 8 pts: 90% (excellent)	
			3-/4-part				−12 pts: 83% (good)	1 iatrogenic radial nerve palsy
			+ shaft: 4				- 3pts: 63% (moderate)	2 nail impingement
							−1 pt: 54% (poor)	(nail removal) (16.7%)
Sforzo [19] 2009	14	56	2-part: 7	40	100%	12 weeks	SPADI: 30 (good)	2 malunions
			3-part: 5				Excellent: 6	(1 retroversion, 1 lesser tuberosity)
			4-part: 2				Good: 2	1 shoulder stiffness & postop complex regional pain syndrome
							Fair: 3	1 loosening of proximal screw (removal) (28.6%)
							Poor: 1 (2 unknown)	
Koike [17] 2008	54	66	2-part: 29	18	100%	Max. 24 months	JOA score: 81 points	1 superficial infection
			3-part: 22				- 79% satisfactory to excellent	4 loosening of proximal screws (7%)
			4-part: 3				- 21% unsatisfactory results	(3 removed, 1 nail removed)
								residual deformities (16%) (9.3%)
Kazakos [16] 2007	27	65.9	2-part: 16	12	100%	Time to sufficient bridging callus on X-rays: 6 weeks	Neer score:	1 screw back-out
			3-part: 11				−6 pts 93.3	1 shoulder stiffness
			4-part: 0				−15 pts 85.4	
							−4 pts 73.5	1 AVN of humeral head (11.1%)
							−2 pts 62.5	
Sosef [20] 2007	28	66	2-part: 16	12	96.4%	not reported	Constant score: 81%	1 non-union
			3-part: 4				-17pts: >75%	1 AVN of humeral head
			4-part: 2				-8pts: 50-75%	4 removal of all or parts of the fixation
			(Metaphyseal extension: 6)				-3pts:<50%	(screw migration into the glenohumeral Joint or impingement)
								3 proximal screw back-out (32.1%)
Agel [14] 2004	20	48	2-part: 16	10	65%	not reported	13 (65%) healed (1 pt died at 5 months with a delayed union)	1 superficial infection
			3-part: 3				−6 were not healed at the time of the last follow-up	1 proximal screw migration into the glenohumeral
			4-part: 1					3 proximal screws loosened
								2 fixation failure (1 revision, 1 healed in varus)
								7 delayed/non-union (70%)
Adedapo [10] 2001	23	68.7	3-part:10	12	-	not reported	Neer score (median):	3 proximal screw loosening & back-out
			4-part: 6				−3-part: 89	1 AVN of humeral head (17.4%)
			3-/4-part				−4-part: 60	
			+ shaft: 7				−3 or 4-part + shaft: 73	
							Constant score (median):	
							−3-part: 91.5%	
							−4-part: 67%	
							−3 or 4-part + shaft: 71%	
Rajasekhar [18] 2001	25^*^	71 (F)	2-part: 23	18	96%	not reported	Constant score	1 non-union
		53 (M)	3-part: 4				-13pts: >75%	1 shoulder stiffness
			(^**^: 3)				-7pts: 50-75%	1 AVN of humeral head
							-5pts<50%	1 proximal screw back-out (16%)

(M: male, F: female, ^*^: initially 30pts, ^**^: not classified).

## Discussion

Proximal humeral fractures occur frequently and when displaced they may require operative treatment. Operative treatment options include open reduction and internal fixation with plates and screws, staples and wires, percutaneous pinning, intramedullary nails, and shoulder hemiarthroplasty [4,6-9]. In the literature, a lack of adequate data regarding evidence-based decision making for the management of displaced or unstable proximal humeral fractures exists [22]. Until now no single operative technique and fixation device has been demonstrated to be superior or without complications [23,24]. The treating physician should keep in mind that the major goal of surgical treatment in displaced proximal humeral fractures is to obtain anatomic fracture reduction and stable fixation in order to minimise pain, promote fracture healing and facilitate early rehabilitation.

We retrospectively evaluated the indications, functional outcome and union and complication rates in patients with displaced and unstable proximal humeral fractures who underwent operative treatment with the Polarus intramedullary nail in our institution within a 2-year period. We acknowledge limitations within our study. Only 2-part, 3-part or 4-part (suitable to nailing) proximal humeral fractures were included; whereas specific fracture patterns, such as head split fractures or fractures extending in the humeral shaft, were not included. Furthermore, the number of cases was relatively small. Nevertheless, our results represent the experience of one single unit. Other strengths of the study include a long follow up and the evaluation of the functional assessment of the patients.

The first published study used the Polarus nail for 2-part and 3-part displaced proximal humeral fractures [18], but its use has been also expanded in 4-part fractures [10]. In the present study, the Polarus nail was also used in 4-part proximal humeral fractures with excellent results (median Constant score: 81) with similar Constant scores to those recorded in patients with 2-part or 3-part fractures (Table 4). We have noticed that the good functional results were mainly obvious shortly after surgery and 12 months post-operatively after intramedullary nailing of 4-part proximal humeral fractures. After that time, no further improvement has been recorder at the follow-up. The short intra-operative time, the limited exposure and minimal soft tissue damage, the preservation of periosteal blood supply and the rapid functional recovery resulted in good functional scores in this group of patients. However, in our study half of the patients with 4-part fracture proceeded to union at a less than 120° of valgus neck/shaft angle, probably due to reduced grip strength of screws at the osteoporotic bone. Yet the radiologic findings did not correlate with the functional scores. Other studies also report excellent to satisfactory results for the same fracture pattern [10,14,17,20,22]. Overall, excellent and satisfactory results with regard to functional outcome (Constant score) was noted in the majority of the patients in this study (92%). Rajasekhar et al. [18] measured a median Constant score of 75 (25–88) points for patients aged over 60 years, and 70 (34–100) points for those younger than 60 years, in 25 patients treated with the Polarus nail. Sosef et al. [20] found a more than satisfactory shoulder function by recording a median Constant score of 89 (range 39–100). Adedapo and Ikpeme [10] treated 23 patients with displaced 3-, 4-part fractures and 3 or 4-part combined with shaft fractures using the Polarus nail and they found a mean Constant score of 88 (40–100), 67 (50–91), and 69 (40–94) points, respectively, at 1-year follow-up. Pain and loss of range of motion were the major reasons for the unsatisfactory results. However, it has been suggested that the pre-injury status of the shoulder seems to influence the prognosis and a coexistent rotator cuff tear or the local rotator cuff damage at the point of nail insertion may jeopardise the functional outcome [24,25]. The later seems highly unlikely, since this incision is cautiously sutured after nail’s implantation [20,26].

From the complications that we have encountered, there were six minor ones (4 backed-out screws, 1 implant impingement and 1 superficial infection), and only one major (4%) including failure of the fixation requiring revision surgery and conversion to shoulder hemiarthroplasty. However, none of the patients developed avascular necrosis of the humeral head, which is one of the major complications after displaced fractures of the proximal humerus [2,24]. The surgical technique for intramedullary fixation harms less the blood supply to the fracture fragments, thus minimising this complication which rates from 0% to 4.34% [10,14,16-18,20]. The overall incidence of AVN of the humeral head was 1.9% within the 215 cases reported in the literature. The frequency of the loosening of the proximal cancellous screws was comparable to other reports, ranging from 3.7% to 15% [10,14,16-18].

## Conclusions

Proximal humeral fractures constitute a significant cause of morbidity in the elderly and the good functional scores achieved with the Polarus nail are promising together with the excellent results in younger patients. Additionally, it can be used in more complex fracture patterns like 4-part with very good results. Other advantages include the short intra-operative time, the limited exposure and soft tissue damage and the preservation of periosteal blood supply. Moreover, it facilitates rehabilitation and it requires short post-operative hospital stay. Union is achieved in 96% of proximal humeral fractures; but a recent study has reported a 100% union rate even when the Polarus nail was used for proximal humerus established non-unions in combination with autologous bone grafting [27].

The overall functional outcome from our case series and the analysed studies is for the vast majority of the cases graded as excellent or good. In elderly patients however, a lower Constant score can be expected. Further studies are desirable to evaluate in more detail its role in 3- and 4-part fracture patterns.

## Competing interests

All authors declare that they have no competing interests.

## Authors’ contributions

FNX, RD and NM contributed to the retrospective collection of the data and the documentation, evaluation and analysis of the clinical, radiological and functional outcome of the patients. They also performed the literature review and writing of specific sections of the manuscript. RH performed the operations and evaluated the clinical and functional outcome of the patients. He was also involved in the interpretation of the data and in drafting the manuscript. PVG has made substantial contributions to the conception of the study and he critically revised the manuscript for its intellectual content. All authors read and have given final approval of the final manuscript.
